# “I forgot she used to make chocolate cake”: Digital storytelling supporting person-focussed dementia care: A qualitative thematic analysis

**DOI:** 10.1177/14713012251317761

**Published:** 2025-01-28

**Authors:** Stephanie Munk, Rhiannon Toohey, Aliaa Remtilla, Nathan M D'Cunha, Diane Gibson, Stephen Isbel, Aisling Smyth, Kasia Bail

**Affiliations:** Centre for Ageing Research and Translation, Faculty of Health, 2234 University of Canberra, Bruce, ACT, Australia; StoryTiling; Centre for Ageing Research and Translation, Faculty of Health, 2234 University of Canberra, Bruce, ACT, Australia; Centre for Ageing Research and Translation, Faculty of Health, 2234 University of Canberra, Bruce, ACT, Australia; School of Nursing and Midwifery, Faculty of Health, 2234 University of Canberra, Bruce, ACT, Australia

**Keywords:** dementia, Alzheimer’s disease, reminiscence, memory, cognition, nursing homes, nurses

## Abstract

Reminiscence is a meaningful activity for people with dementia, but research implementing digital reminiscence tools into environments with older people is not well developed. This project sought to understand the effectiveness of a digital reminiscence tool in aiding person-centred dementia care with people attending a day respite centre and a group residential home, in metropolitan eastern Australia. This study used semi-structured interviews and ethnographic observations using a qualitative reflexive thematic analysis with seventeen participants including people with dementia (*n* = 8), their loved ones (*n* = 5) and staff *n* = 4) Themes identified were: 1. Remembering myself; 2. Reminiscing the person before and with dementia; 3. Enhanced relationships through self-expression; 4. Person-centred adaptation and the art of the interview; and 5. Future potential. Findings indicate that the StoryTiling app was user-friendly, supported reminiscence activities and enhanced person-centred care. The reminiscence activity enhanced relationships between participants, families, and carers, facilitating a deeper knowing of the person with dementia. The activities supported positive memories and emotions and helped reinforce the identity of the person with dementia in both their own mind, and their loved ones. The information captured in the StoryTiling process enabled person-centred care in improving the ability to know a person and being able to relate and respond to their individual needs, wants and goals. The process was dependent on the ‘art of the question’ and the ‘art of the interview’, particularly by people who know the person with dementia and are trauma-informed in order to effectively progress interviews and utilise them within the care environment. Enabling nudge activities that promote person-centred engagement such as reminiscence through digital storytelling may help foster person-centred care in the aged care sector.

## Background

The experience of living with dementia varies greatly as no two individuals experiences the same disease progression, with both positive and negative emotions reported (Górska et al., 2018). Reductions in the ability to effectively communicate and maintain relationships can contribute to a sense of loss and uncertainty with associated anger (Mazaheri et al., 2013) and feelings of isolation (Van Wijngaarden et al., 2018). Positive emotions such as love, gratitude, happiness, and optimism are also reported (Karlsson et al., 2014) and supported by ongoing involvement in activities, structured routine incorporating flexibility, and maintaining independence and social connectivity (Berglund et al., 2019; Karlsson et al., 2014). Achieving these positive outcomes is enhanced by a person-centred model of care, and is considered essential for people living with dementia (Berglund et al., 2019).

Person-centred care is described as the personalisation of health care, focusing on the person as central in the design and delivery of services (McCormack et al., 2015; Phelan et al., 2020). Inherent in a person-centred model of care is knowledge of, and respect for the person as an individual with unique life experience, history and culture, likes and dislikes (King & Miller, 2021). Person-centredness is associated with a range of positive outcomes, including improved patient safety, quality of care, quality of life, mental health and well-being and mortality (Chenoweth et al., 2022; Fazio et al., 2018; Martin-Khan et al., 2020; Meterko et al., 2010; Tsai et al., 2015). Using a person-centred approach can improve identification of symptom changes during the deterioration of health, due to familiarity with the person’s baseline or ‘normal’ state and increased sensitivity to variation in personality and expression, which is often the first sign of deterioration such as in delirium (Yevchak et al., 2017).

People with dementia live and receive care in a variety of settings including community, residential aged care, hospital, and respite care (AIHW, 2021b). When hospitalised, people with dementia are more likely to experience higher rates of complications such as pneumonia, delirium and urinary tract infections (Bail et al., 2013), have rationing of fundamental care such as communication and mobilisation (Bail & Grealish, 2016), experience a lack of workplace flexibility (Harmer & Orrell, 2008) and a task-focussed rather than person-focussed approach exacerbated by insufficient staff numbers (Gibson, 2022). Together, these factors are often barriers to supporting person-centred care practices, with consequences for staff as well as people with dementia.

Compassion fatigue, psychological distress, burnout, ethical and moral distress have been frequently reported among the aged care workforce (Jameson & Parkinson, 2022; Jones-Bonofiglio, 2020; Midtbust et al., 2022). Coping mechanisms for psychological distress can paradoxically involve adopting a more task-oriented approach (Jones-Bonofiglio, 2020; Midtbust et al., 2022), however this is also associated with burnout (Westermann et al., 2014) and poor staff retention (Barbosa et al., 2015) which further reduces the quality of care (Branson, 2019) and contributes to a cycle of avoidance of person-centred care. Workforce psychological distress can be ameliorated by empathy-focussed or person-centred care (Alhalal et al., 2020; Chang et al., 2020; Yevchak et al., 2017). Consequently, implementing strategies to sustain person-centred care will benefit staff and in turn residents.

There is positive evidence that a range of interventions targeting meaningful engagement, including massage, exercise, aromatherapy, pet therapy and music therapy (Bessey & Walaszek, 2019; Brasure et al., 2016; Livingston et al., 2014) can support wellbeing, psychological needs, belonging and identity (Harmer & Orrell, 2008), and reduce stress and improve memory for people living with dementia (Hu et al., 2018; Kales et al., 2019; Lamont et al., 2018). Success of these interventions is improved by ensuring they are individualised (Harmer & Orrell, 2008). One key meaningful activity that is inherently individualised and person-centred is reminiscence therapy.

Reminiscence therapy involves capturing life stories and relates to a persons’ past roles, practices, and activities (Harmer & Orrell, 2008; O'Philbin et al., 2020; Woods et al., 2018). Reminiscence activities provide positive, person-centred experiences for carers and families of people with dementia (Doran et al., 2019; Ervin et al., 2013; Karlsson et al., 2014). Potential benefits include enhanced care, assistance with navigating confusion, finding safety and maintaining a sense of identity and dignity (Heggestad & Slettebø, 2015), strengthened relationships (Ervin et al., 2013; Karlsson et al., 2014), assistance with improving quality of life and communication (Subramaniam & Woods, 2010), and improvements in cognition and mood (Ching-Teng et al., 2020; Cotelli et al., 2012; Travers et al., 2016). Reminiscence therapy, as a meaningful activity, allows a deeper knowing of the person with dementia and permits activities to be designed around them and their abilities, personality and interests (Clune et al., 2023). Previous research by Woods et al. (2018) highlight uncertainties around clinical implications of reminiscence therapy due to inconsistencies in research such as different contexts (e.g.: community or home), types of reminiscence activities, and differences in participants (e.g.: group or individual), recommending further investigation.

Traditional reminiscence therapy consists of paper-based books, conversation prompts, and reminiscence kits made up of physical artifacts. Examples include audio recording of a person’s life story which is transcribed and published in book format (Beyond Words - Celebrating Life Stories), to downloadable templates to capture life stories (For example, Anderson, 2022; Clinical Excellence Commission, 2014).

In recent years, reminiscence therapy has evolved, with the integration of technology resulting in an increasing availability of digital RT platforms. Current digital RT range in formats including electronic platforms to help care staff in creating stimulating conversation (e.g. ‘Book of You’); and photo, music, audio and video platforms designed for people with dementia (e.g. InspireD). Other examples focus on written stories (e.g. StoryWorth, Storii), audio stories (e.g. Trysaga), or are subscription-based funded models (e.g. lifebookuk.com costs $AUD12,000; alastingtale.com costs AUD$1299; nostorylost.com costs 800USD). Recent developments include immersive reminiscence therapy experiences such as ‘reminiscence rooms’ (Goodall et al., 2021) and virtual-reality embedded reminiscence activities (Huang & Yang, 2022).

Numerous reviews have found that people living with dementia benefitted from digital reminiscence therapy through improved mood and cognition, increased social interaction and an ability to take ownership of the conversation (Astell et al., 2018, Grossberg et al., 2021, Lazar et al., 2014; Pappada et al., 2021). Moreover, the delivery of digital reminiscence, when compared with traditional, has some key benefits including the ability to instantaneously access a rich database of salient artifacts, increased accessibility via potential adaptions to address motor or sensory impairments (magnification of artifact images, use of headphones, touch screen navigation) and increased ease of therapy delivery (Lazar et al., 2014). Key barriers identified were the technological requirements and costs for set up, inhibiting application with care delivery practices (Lazar et al., 2014).

Given the size of the growing population of people with dementia, their families, carers, difference in service providers settings, workforce, culture and education, digital technology could help reminiscence activities realise its potential as an accessible, adaptable, scalable, and cost-effective intervention to support person-focussed dementia care. This research aimed to explore a free digital reminiscence tool in aiding person-centred dementia care with people attending a day respite centre and/or a group residential home in Canberra, Australia.

## Method

### Background

**Figure 1.**
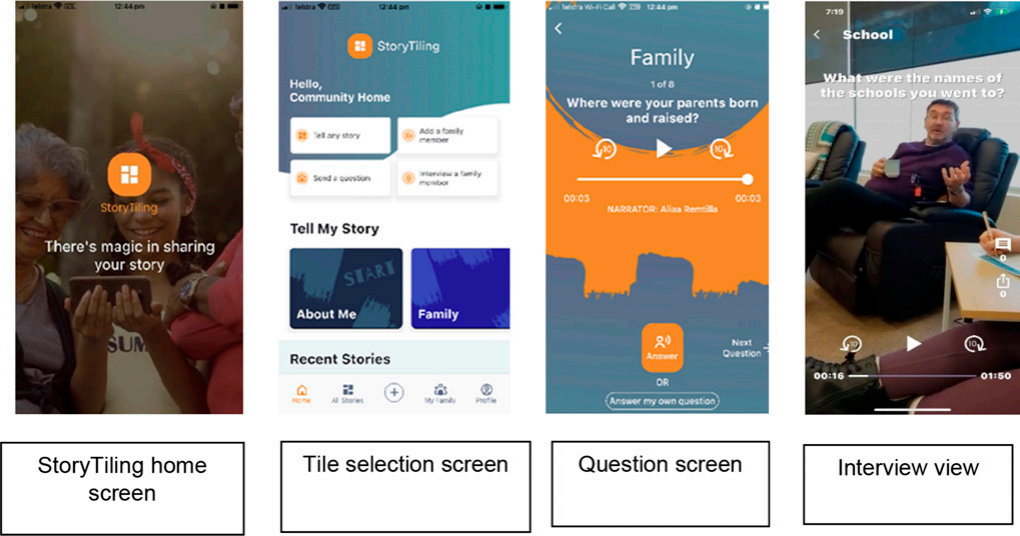
StoryTiling screenshots.

StoryTiling was created in 2021 and is a digital application (app) that uses anthropological science to help people record their family stories. See Figure 1 for a visual overview of how the app presents.

StoryTiling is freely available on multiple platforms (e.g. android, iPhones) and provides prompts to elicit stories which are recorded in video format. Prompts within the StoryTiling app are categorised in topic tiles such as family, career, hobbies, and travel. On early discussions in relation to the project setting and aim, the developers added a new tile for the purposes of the project, the ‘Person-centred tile’. Each topic tile, when selected, opens further questions. For example, within the ‘family’ category, questions may include “where were your parents born and raised?” or “what characteristic of your mother do you most admire?”. The person-centred care tile included: “what are the things to remember about me?”; “what do people say about me?“; What is important to me?“, “how to best support me?“. The recordings can be shared by sending a link to the recording that can be viewed outside the app.

### Study design

This study used a constructivist enquiry approach towards the qualitative research methodology in the form of semi-structured interviews and ethnographic observations to support reflexive thematic analysis (O'Connor, 2001; Braun & Clarke, 2019). This research received ethics approval from The Human Research Ethics Committee of the University of Canberra (UC HREC # 11918).

### Settings

The research took place at two sites, a small-scale residential group home (and a day respite centre that serve people with mild to moderate dementia with a focus on younger onset dementia. The small scale residential group home is located on a suburban street and houses 6 people (guests). It operates with a ratio of 1 staff member (or buddy) to 3 guests, Guests of the residential homes may also attend, an inclusive day respite centre providing activities for people (members), supported by staff (buddies), where family members (loved ones) can also attend. The setting is a large open-plan area with various sections devoted to specific activities. Most sessions and interviews were conducted in quiet areas of the day respite centre.

### Participants

Inclusion criteria were: residents of small scale residential home (guests; *n* = 5), and people who visited the day respite centre (members; *n* = 3) in the first half of 2023; informal carers of members or guests (loved ones; *n* = 5); formal care staff (buddies; *n* = 4) (Table 1). Medical information was provided by the organisations registered nurse, and diagnosis was simplified to ensure nobody could be re-identified. All staff had been working at the organisation since the opening less than two years prior.

**Table 1.** Overview of participant characteristics.

Participant Type (*n* = 17)	Pseudo-nym	Gender	Age Range	Diagnosis or relationship to person with dementia	Length of residence	Interview	Observation
Guest	Michael	Male	56–65	Dementia, diabetes	15 months	No	Yes
Guest	Bruce	Male	66–75	Dementia, cancer	14 months	No	Yes
Member	Carol	Female	76–85	Parkinson’s disease Dementia	6 months	Yes	Yes
Member	Rosa	Female	66–75	Multiple sclerosis, dementia, osteoarthritis	10 months	Yes	Yes
Member	Stainless	Male	56–65	Dementia	6 months	Yes	Yes
Guest	Bill	Male	66–75	Dementia, diabetes Tachycardia	9 months	Yes	Yes
Guest	Polly	Female	56–65	Alzheimer’s disease	12 months	Yes	Yes
Guest	Anne	Female	66–75	Alzheimer’s disease	21 months	Yes	Yes
Buddy	Isobel	Female	56–65	Staff member	N/A	Yes	Yes
Buddy	Sarah	Female	16–25	Staff member	N/A	Yes	Yes
Buddy	Scarlet	Female	35–45	Staff member	N/A	Yes	No
Buddy	Vara	Female	46–55	Staff member	N/A	Yes	Yes
Loved one	Rupert	Male	76–85	Loved one of carol	N/A	Yes	Yes
Loved one	Dylan	Female	66–75	Loved one of bill	N/A	Yes	Yes
Loved one	Ben	Male	66–75	Loved one of polly	N/A	Yes	Yes
Loved one	Bob	Female	36–45	Loved one of Anne	N/A	Yes	Yes
Loved one	Tracey	Female	36–45	Loved one of Rosa	N/A	Yes	Yes

Member = person with dementia at respite care; Guest = person with dementia residing at small scale residential group home; Buddy = staff member working with people with dementia; Loved one = informal carer of person with dementia.

### Recruitment

The research project was advertised via paper flyers in prominent locations and digitally via WhatsApp and email l for loved ones and staff. Information about the project was presented to individuals at the day respite centre with information sheets, including the voluntary nature of participation, option of withdrawal, and confidentiality. Individuals were able to have questions answered and complete consent forms where appropriate. Collaboration occurred with a Registered Nurse (RN) who assessed a prospective participant’s capacity to give consent before written consent was requested from the prospective participants’ and/or their Enduring Power of Attorney (EPOA).

### Implementation

The implementation approach was developed in conjunction with the staff, members and loved ones, with the purpose of reminiscence therapy outlined, and the approach to safeguard participants developed. Members with dementia and loved ones were invited to early information sessions, and supported the development of the approach to trial the app. A StoryTiling account or profile was consequently created forthe organisation and guests/members were added as family members. This enabled the participant to have their own profile, where all recordings were automatically added. Staff provided with the log in details were then able to see any of the recordings and/or create more. The log in details were not shared with anyone outside of the organisation due to the privacy and confidentiality of multiple guest/members being on the one account. The app has functionality to share a link to a video with an external person, allowing them to view that video only. Links to some videos were shared with loved ones at the request of loved ones and they received the specific links to their person only. Loved ones were given the option of creating their own account and therefore doing their own interviews, but no one chose this option.

Video length varied greatly, depending on the participant. Participants were given the opportunity to view their videos, and more videos could be recorded and shared via a link as described. The organisation were intending to continue using the app and thought it would be particularly useful for new staff, allowing them to get to know people very quickly thus supporting quicker integration of person-centred care.

Participants and loved ones were given a brief overview of the StoryTiling app at the initial information session with links provided for them to explore if they wished to. The person asking the questions from the app was given a brief demonstration of how to use the app before commencing and was able to be guided by the observer if any difficulties with the app were encountered.

### Data collection

Ethnographic observations occurred whilst the StoryTiling sessions were being recorded, and field notes were written during or directly after each session. The observations consisted of events and interactions including the setting, the approach used, the participant’s presentation, direct quotations, the researcher’s reactions, and anything that occurred during StoryTiling interviews. The notes were revised later for their comprehensiveness and further details added when necessary, supporting the rigour criteria of dependability (Forero et al., 2018). This style of ethnographic observation is a kind of ‘immersive relational practice’ to develop understandings and knowledge (Pilbeam et al., 2023).

**Figure 2.**
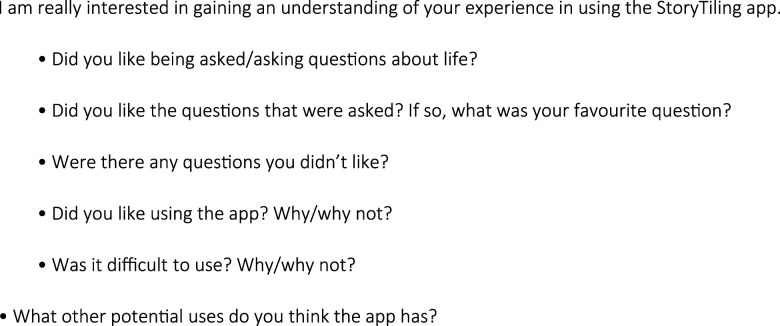
Participant Interview prompts.

Semi-structured interviews were conducted by asking open-ended questions (see Figure 2) to the guests/members, buddies, and loved ones about the StoryTiling application. The interviews were recorded and transcribed for analysis. Interviews were conducted at a time and location convenient to the participants in the days or week after the StoryTiling session (O'Connor, 2001). A flexible approach to the semi-structured interviews was used, which enabled participants to lead the direction of the conversation. Any questions asked during or after the interview were promptly addressed (Mills et al., 2006).

Data were collected by two researchers (SM, RT). One of the researchers was also a ‘Buddy’ at the organisation. This circumstance is referred to as ‘insider research’, which occurs within a group or organisation in which the researcher is also a part of (Fleming, 2018). To balance the dual positions of carer and researcher, the staff member contributed to writing of the ethics proposal, and continually reflected in team discussion regarding potential conflicts and bias. The dual role supported the project and analysis, in that pre-established relationships with the participants with dementia meant ability to adapt questions to their individual needs more easily due to being privy to their life history, mannerisms, and level of ability (Chammas, 2020). By having a familiar person asking them about their lives, the participants also appeared to be more relaxed and at ease with sharing personal information about themselves, allowing for richer information to surface. This position was further applied through the analysis phase, allowing for different insight related to the participant experience based on carers specific knowledge of the participant.

### Data analysis

Reflexive thematic analysis was used to explore the participant experiences in this study. This method allowed the researchers to actively engage with the data while also considering their own position and influence on the research process (Braun & Clarke, 2019)**.** An inductive approach was used that involved working exclusively from participant experiences to drive the analysis of the interviews and ethnographic qualitative data (Thomas, 2006) with three members of the research team: an experienced registered nurse researcher (KB), a registered nurse research assistant (SM), and a staff member and the ‘insider researcher’ (RT). The researchers used an iterative process in which texts were read multiple times to acquire a detailed view of how the StoryTiling application was perceived by participants. Following multiple readings, segments of text were coded into initial thematic groupings, supported by analytic memos and discussions developing theoretical ideas in relation to the research questions (Azungah, 2018). Collation of the coded data was analysed inductively using theoretical and contextual knowledge of the researchers (Thomas, 2006). Meaning was created about each interpreted point creating a reflexive thematic analysis finding patterns of shared meaning throughout the data (Braun & Clarke, 2019). Member checking was used to provide an opportunity to validate the findings and ensure that they are grounded in the perspectives of the participants, supporting credibility and rigour (Braun & Clarke, 2019; Lincoln & Guba, 2007). Their comments were incorporated back into the analysis. The findings were also presented to the app developer (AR) who contributed to late-stage presentation of the themes. Findings are presented with the use of direct quotations supporting transferability and confirmability (Lincoln & Guba, 2007; Nowell et al., 2017).

## Results

### Study overview

Seventeen participants were recruited including five guests, three members, four buddies and five loved ones. Characteristics of the study participants are outlined in Table 1. A range of StoryTiling session approaches were completed as detailed in see Table 2, with time spent on StoryTiling ranging from 24-54 minutes and the number of stories recorded per participant ranging from 4-26 stories. All StoryTiling sessions were done in a single session, the length of time dependent on the participant interactivity and perceived comfort by the interviewer. A total of eight ethnographic observations were completed, and ten interviews.

**Table 2.** StoryTiling session characteristics.

Pseudonym	Ethnographic identifier	Approach *	StoryTiling Tiles used	Time spent on tile	Number of stories recorded	Total time
Ben and polly	1	3. Guest/member with a loved one (+/− buddy support)	Travel	16 mins	26	40 mins
			Career	10 mins		
			First love	4 mins		
			Family	6 mins		
			Person-centred care	4 mins		
Bruce	2	1. One on one (researcher and guest/member)	About me	15 mins	13	40 mins
			Travel	9 mins		
			Family	16 mins		
Bill, dylan and Vara	3	3. Guest/member with a loved one (+/− buddy support)	About me	5 mins	24	24 mins
			Family	6 mins		
			Life partner	6 mins		
			Travel	4 mins		
			Person- centred care	3 mins		
Stainless and sarah	4	2. Guest/member with a buddy	Family	22 mins	7	54 mins
			Travel	30 mins		
			Career	2 mins		
Michael and Isobel	5	2. Guest/member with a buddy	School	17 mins	8	44 mins
			Person-centred care	12 mins		
			Travel	15 mins		
Michael and Isobel (in a group)	6	4. Group setting- members and buddies	Career	17 mins	4	22 mins
			Sport	5 mins		
Tracy and Rosa	7	3. Guest/member with a loved one (+/− buddy support)	Family	5 mins	19	24 mins
			About me	4 mins		
			Career	3 mins		
			Hobbies	2 mins		
			Person- centred care	10 mins		
Carol and Rupert	8	3. Guest/member with a loved one (+/− buddy support)	Family	14 mins	21	40 mins
			Travel	6 mins		
			Life partner	10 mins		
			Person- centred care	10 min		

Five themes with associated subthemes were generated from analysis of the ethnographic observations and interviews (Table 3). The five themes were (1) Remembering myself; (2) Reminiscence forthe person before and with dementia; (3) Enhanced relationships through self-expression; (4) Enhanced relationships between buddies and members and (5) Enhanced relationships with other members and guests. Supportive quotations are presented according to Themes and Subthemes in Table 4. Names are pseudonyms which participants selected. [Square brackets] are used to notate researcher insertion of word and ellipses […] and **bold** text to aid readability.

**Table 3.** Themes and subthemes.

Themes	Sub-Themes
**Remembering myself**	a. Reminiscence was a positive experience b. Revisitation of central topics helped revise memories c. Prolonged and repeated reminiscence was valued
**Reminiscing the person before and with dementia**	a. Memory, emotion and identity retention b. Remembering the person before dementia c. Emotional response to the interview d. StoryTiling enables a reflective space for loved ones
**Enhanced relationships through self-expression**	a. Enhanced relationships between loved ones and members b. Enhanced relationships between buddies and members c. Enhanced relationships between members/guests
**Person-centred adaptation – the art of the interview and trauma informed care**	a. The art of choosing questions b. The art of knowing the person to prompt the conversation and following the person’s lead
**Future potential**	a. Use as a handover tool to support person-centred care b. Updated visualisation - creating new memories by recording old memories c. Intergenerational uses d. Suggested changes

**Table 4.** Quotations and ethnographic notes supporting Themes and SubThemes.

1. Remembering myself	
**a. Reminiscence is a positive experience for identity**	*‘I miss going with those guys I used to work with’ (Michael, guest)*
	‘*[Carol said] ‘I’m going to go to Europe, buy a motorbike and ride it all the way home.’* (Rupert, loved one) ‘*Oh my god yes…*. *I* ***did*** *buy…. a motor scooter*.’ *(Carol, member)*
**b. Revisiting central topics helped revise memories**	*[Michael] crying out of love for his brother, demonstrating deep feelings (Ethnographic notes 6.)*
	*However, the different questions prompted him to speak of different aspects of his family, such as their ages and religious orientation (Ethnographic notes 2.)*
**c. Prolonged and repeated reminiscence lasted beyond the story tiling sessions**	*Stainless showing anger and sadness, talking about his journey developing dementia ‘* ***I was*** *a sports trainer.’ (Ethnographic Notes 4)*
	*Travel leading into talking about fishing and family members (Ethnographic notes 2.)*
	*Keeps reverting back to telling stories about family (Ethnographic notes 2.)*
	*Ben explains the impact family had on Polly and her life as well as their relationship (Ethnographic notes 1.), relaying how important family is to Polly*
2. Reminiscing the person before and with dementia	
**a. Memory, emotion and identity retention**	*‘It’s good to have you here with her. Because when you tell a story, and [Carol] can listen and remember.’ (Carol, member)* *‘After he tells the story do you remember it then?’ (Researcher)* *‘Yes, yes it helps.’ (Carol, member)*
	*Telling the same story […] different details are being added to the story (Ethnographic notes 1.)*
**b. Story tiling enables a reflective space for loved ones**	*[What do people say about Rosa?] ‘Full of energy, just go go go. Which is why this illness has been difficult to accept.’ (Tracey, loved one)*
	*To bring up all these things that have been having and to think about all the things that we have talked about over the years…. remember things I hadn’t thought about for a long time (Dylan, loved one)*
	‘*His [Bill’s] mum was always, sort of world famous for cooking brilliant scones… They’d be on the table before the kettle was boiled. [laughs]*’ *(Dylan, loved one)*
	*‘It just brought back memories of all the things she used to do before she got sick, which was nice’. [Which made me feel both] happy and sad. I mean it’s been 12 years since she last baked a chocolate cake for me. So just memories of the baking, the cooking and watching foreign international movies. I’d forgotten, you know? You forget after so many years what we used to do’ (Tracey)*
3. Enhanced relationships through self-expression	
**a. Enhanced relationships between loved ones and members**	*‘She was strong and active’ (Tracey, loved one), showing pride again for her mother (Ethnographic notes 7.)*
	*Tracey rubs Rosa’s arm and smiles at her lovingly when walking and talking about how amazing she was at dancing as she travelled around the world performing. (Ethnographic notes 7.)*
	*‘And what does he do? (Referring to their son)’ (researcher)* *‘He works at the Australian Bureau of stats.’ (Ben, loved one)* *‘Oh, very smart.’ (researcher)* *‘He was yeah, very clever. But he had his mother’s [Polly’s] work ethic.’ (Ben, loved one)* *‘Sure, very hard worker?’ (researcher)* *‘As (Polly) was, yeah.’ (Ben, loved one) (Ethnographic notes 1)*
	*I know when I was away with Patrick. We talked about different things. And some of them he’d never heard before, just because we had time to actually sit down and talk to each other. (Dylan, loved one)*
	*So just memories of the baking, the cooking and watching foreign international movies. I’d forgotten, you know? You forget after so many years what we used to do.’ (Tracy, loved one)*
	*‘[I saw] a really cool video of mum actually interacting with that projection table, which was so nice for me to see. Because I always think oh, is she just sort of like this [mimics person lying static]. So, to think that she’s actually interacting is nice.’ (Tracy, loved one)*
**b. Enhanced relationships between buddies and members**	*‘You get to learn more about that person and what they’ve done in their life’ (Isobel, buddy)*
	*‘I learned lots of little things about the members that I didn’t know, stories that I hadn’t heard before. Things about their life, things that they love.’ (Scarlett, buddy)*
	*‘I read the stories on the app to better get to know my members.’ (Scarlet, buddy)*
	*‘I didn’t know as much about [Stainless] until I did that interview, so it was good.’ (Sarah, buddy)*
	*‘I just know more about him, like I feel like I haven’t asked those specific questions before […] the things we found out were like very important. Like who he is. …. To know how to support him and like what kind of things he likes to do and like to know him better. Yeah, just to know who he is and then like what he would like to do.’ (Sarah, buddy)*
	*‘So whenever […] have to see more people then I will get more information about them. And […] it will be more easier for me while doing the job.’ (Robert, buddy)*
	*Buddy relating to the story being told by sharing personal information. (Ethnographic notes 1)*
**c. Enhanced relationships with other members/guests**	*Well it’s fun, It’s always fun talking about one’s self. A life of no great significance but it’s nice to have somebody appear to be interested. (Rupert, loved one)*
	*Empathetic responses from other guests/members [when hearing a story] (Ethnographic notes 6.)*
	*Participant speaking to the whole group [telling their story in a group setting], not just the buddy (Ethnographic notes 6.)*
	*Another member chiming in to help when filling in the info on the story (Ethnographic notes 6.)*
	*Other guest/member leaning in to listen to the story (Ethnographic notes 6.)*
	*‘He won a grand final’ (Stainless, member) [relaying something he learnt in a previous session]. “He did” (Sarah, buddy)*
4. Person-centred adaptation – the art of the question and trauma informed care	
**a. The art of choosing questions**	*Instead of asking ‘what is your favourite memory of your mum?’ The researcher said: ‘Do you know what (Polly’s) favourite memory of her mum is? Or a moment that you might have shared?’ (Ethnographic notes 1)*
	*Rosa [is] not able to fully answer for herself so Tracey assists (Ethnographic notes 7). ‘Do you have a favourite memory of your dad?’ (Tracey, loved one) ‘Yeah.’ (Rosa, member) ‘What was he like?’ (Tracey, loved one)*
	*Rupert tries to prompt Carol’s memory ‘Where did you live?’” (Ethnographic notes 8.)*
	*‘Questions were very detailed, and I thought they were not relevant, so they were skipped’ (Bob, loved one)*
	*Buddy scrolls through questions trying to find ones that would relate to Michael (Ethnographic notes 5)*
**b. The art of knowing the person to prompt the conversation and follow the person’s lead**	*Tracey suggests an answer and Rosa repeats it in confirmation but does not answer for herself. ‘Where were your parents born and raised’ [interviewer question] ‘So one was born in Albania, was it?’ (Tracy, loved one) ‘Yeah’ (Rosa, member), ‘And one in Bosnia’ (Tracey, loved one), ‘In Bosnia’ (Rosa, member) (Ethnographic notes 7)*
	*Ben talking about Polly’s sporting and teaching career, [including Polly when she walked over] ‘She was more interested in family life at that time. [Polly approaches] Weren’t you, hey?’” (Ethnographic notes 1)*
	*[…] Bill not answering questions but agreeing when asked yes/no questions. ‘You like cats Bill?’ (Vara, buddy) ‘Yep’ (Bill, member)” (Ethnographic notes 3)*
	*Rosa replies in her first language and Tracey interprets for her. ‘He used to travel a lot and then he brings back presents’ (Tracey translating for Rosa). (Ethnographic notes 7)*
	*Vara substituted the question to apply it to the situation with loved one. (Ethnographic notes3)* *Instead of reading out the provided question, ‘how did you meet your loved one’, Vara changed the question to read: ‘So (Dylan), how did he (Bill) meet the love of his life?‘* *This is because Vara knew that Bill had a previous marriage before Dylan became his wife. By doing this Vara was making a person-centred adaptation to the question by being sensitive to the loved one’s history and experience*
	*Using prompting questions well that related to Bruce and his mother and brothers and sisters (Ethnographic notes 2)*
	*When the loved one or member didn’t know how to answer to the initial question, the Buddy generalised it to still gain meaningful information on the topic (Ethnographic notes7)* *‘What was your first full time job?’ (researcher)* *‘Oh, we wouldn’t know that.’ (Tracey, loved one)* *‘Or what did you do for work? (To Rosa)’ (researcher)* *‘Back home, in the city where mum [Rosa] was born, both mum and dad worked for an energy company.’ (Tracey, loved one)*
	*Use initial question as prompt and follow with leading questions from the answer given. (Ethnographic notes 4)*
	*Buddy asks the loved one questions about her siblings when Rosa couldn’t answer for herself about her children (Ethnographic notes 7)*
	*‘What came up in the question or what you ended up talking about wasn’t actually what the question asked.’ (Sarah, buddy)*
	*Buddy asking follow up questions related to the initial question. (Ethnographic notes 4.)* *‘You came straight to Canberra area?’ (Sarah. Buddy)* *‘Yep. And that was because my father, and some aunties and uncles said to mum and dad, you’ve got to come to Australia, it’s terrific it’s a lot better than what we’ve got here [In Scotland]*.’ *(Stainless, member)*
	*Buddy suggests words to fill in blanks when Stainless gets stuck. (Ethnographic Notes 4.)*
	*Questions act as a prompt for when the story gets too far off track. (Ethnographic notes 5*)
	*Buddy assisting where they think is needed by prompting with new question. (Ethnographic notes 4)*
5: Future potential	
**a. Suggested changes**	*‘They were good [questions] but had to give lots of prompts as Ann remembers so little from the past’ (Bob, loved one)*
	*‘I had to rephrase a lot, based on the knowledge of Ann because I knew she wouldn’t know the answer [due to complexity] (Bob, loved one)*
**b. Use as a handover tool to support person centred care**	*‘[…] it’s helpful for doctors or geriatricians […]’ (Isobel, buddy)*
	*‘I hope it does end up being utilised if not in hospital, in aged care facilities or a care setting…[it would help in] the chaos and having to explain why she’s shouting or this or that or the other.’ (Tracy, loved one)*
**c. Intergenerational use**	*I know when I was away with [ my son] we talked about different things and some of them he’d never heard before. Just because we had time to actually sit down and talk to each other.’ (Dylan, loved one)*

## Theme 1: Remembering myself

People with dementia highlighted the reminiscence as a positive experience that helped revise memories, including re-telling of stories and revisiting topics in the following days after the StoryTiling session.

### Subtheme a: Reminiscence was a positive experience for identity

The participants displayed diverse emotions such as simple enjoyment, deep love evoking tears, mild sadness due to missing someone, happiness when talking about their grandchild, pride for their past achievements and anger at what had been lost due to experiencing dementia.

‘I quite enjoyed the questions. It makes me think about the past and remember it a bit more.’ (Carol, member).

The StoryTiling session was seen to create an emotional response, with the supported reflections about life seeming to reinforce perceptions of self-identity, whether that was based on being an adventurous traveller of the world or a national league footballer.

‘I am very proud of playing in a grand final’ (Bruce, member)

Whilst sometimes the member couldn’t remember or answer the question, often their loved one telling the story for the member would prompt the member’s memory, who would then add details and perspective to the story. The member being reminded of stories from their lives in a supported manner seemed to impact in recreating memories, preserving their sense of identity and worked to prompt access to new memories.

‘You used to tell stories about your father…Her dad came back from the war. Can you tell the rest of the story?’ (Rupert, loved one)

‘Um...No, I've forgotten. Lucky I’ve got my memory (referring to Rupert).’ (Carol, member)

‘She was looking forward; I mean the whole family was.’ (Rupert, loved one)

‘We were excited.’ (Carol, member)

‘Yes, everybody’s excited that dad’s coming back. And he was sort of grumpy and didn’t give the kids a cuddle or anything and didn’t have any presents. It was a big disappointment. Am I right?’ (Rupert, loved one)

‘Yes, you’re right. He had seven sisters, and he was the only boy. My mother’s brother was nicer.’ (Carol, member)

### Subtheme b: Revisitation of central topics helped revise memories

Throughout using StoryTiling, participants would repeatedly mention common topics and stories. For many of the participants, the repeated stories were those relating to family. Questions asked about other areas of a person’s life often led back to this theme.

For Bruce (member), this [repeated topic] was family. It was clear that family is so central to his life as he found a way to bring it up under almost every tile, such as travel and passions/hobbies. (Ethnographic notes. 2.)

The revisited topics appeared to be important or central in their lives, as they were a salient point in their memory.

‘I was put back by the poorness of the rest of the world. We all have it real easy I'll tell you.’ ….’ But I’m not [a churchgoer] no more. I put it down to the poor people in the world I guess…… The eldest bloke [me] jumped out [of being a church goer] and then the rest of them jumped out after him [referring to his children].’ (Bruce, member)

For example, the travel tile discussion with Bruce triggered memories about how some people experience poverty, which he then linked to his loss of faith, and his children’s changed views on the church. His linkages highlight the reflective thought processes for Bruce participating in reminiscence and solidifying his central concepts of family.

However, other family members highlighted that while sometimes StoryTiling sessions could trigger memories, oftentimes they did not.

‘Yes, I think that they were interesting and succeeded in triggering a bit of [Carol's] memory. But as you noticed they didn't always, and many of them she just had to say I don't remember and that was perhaps the most common response I think.’ (Rupert, loved one)

Despite this, family members highlighted that the storytelling was useful even if the person with dementia didn’t talk because they were exposed to the memory and therefore this may trigger them to think about the memory, even if not participating in discussions. Examples were noted of when incidents highlighted key memories that triggered an exultant response associated with the memory

Because we just talked about where we first met, and I just mentioned the place still remember that?’ (Rupert, loved one)

‘Curious Cove!’ (Carol, member)

The reinforcement of core memories shared between loved ones and people with dementia was a valued experience by participants.

### Subtheme c: Prolonged and repeated reminiscence lasted beyond the StoryTiling sessions

After completion of the StoryTiling session, participants would often continue to recall additional information about their lives they had not mentioned during the session. Participants continued to reminisce and then talked further about those additional memories with others in the hours and days after the StoryTiling session. By bringing up those initial memories the participants continue to reflect and then also communicated aspects of their life history.

‘We forgot to mention a lot of countries [that we travelled to] like Germany and Austria and Hungary.’ (Tracey, loved one)

Often participants would re-tell the same story during the StoryTiling session, but with each revisiting the memory added more depth and detail to the story/memory. This finding demonstrates a potential cognitive benefit in that the individual has continued to process the memory and additional details are forthcoming on repeat of the conversation, which were most often initiated by the individual.

## Theme 2 Reminiscence for the person before and with dementia

### Subtheme a: Memory, emotion and identity retention

Loved ones found StoryTiling useful for reminiscence and could recall shared moments between themselves and the participant. This appeared to elicit fondness between the loved one and the participant.

‘There was one time when we were in Budapest, in Hungary, when this is before the, before the iron curtain came down… [but then] I realised I left my wallet on my passport in the bedroom. Couldn’t get back into the building. (Polly) just shook her head, because I used to do that sort of stuff all the time.’ (Ben, loved one)

These emotional connections seemed to foster positive emotions and reinforce the shared memories of the shared life with the person with dementia.

‘It makes you feel good especially when you know, talking about stuff like, how you met somebody. Yeah, good memories.’ (Dylan, loved one)

Warmth and laughter were emphasised and valued in this shared memory of a person’s identity..

‘Did you have a favourite question?’ (Researcher) ‘Probably, what do people say about [Bill]? That's always good to remember you know, because it's hard sometimes when you see him like he is now to remember what a special guy he was.’ (Dylan, loved one)

These positive feelings associated with the stories could be seen to reinforce the concept of the person with dementia and their place within the social context, thus reinstating their personal identity, before dementia.

### Subtheme b: StoryTiling enables a reflective space for loved ones

In remembering what the person was like before their illness, emotions such as joy, happiness, and love were expressed, but also feelings of sadness, loss, guilt, anger and regret.

‘Did you like being asked questions about your life and about [Rosa’s] life? [Researcher to Tracey]

‘I did, yes, it just brought back memories of all the things she used to do before she got sick, which was nice’ [Tracey]

‘How did that make you feel’ [Researcher]

‘Well happy and sad. I mean it's been 12 years since she last baked a [chocolate] cake for me. So just memories of the baking, the cooking and watching foreign international movies. I’d forgotten, you know? You forget after so many years what we used to do’ [Tracey]

In turn, this space enabled loved ones to consider and extrapolate some of the complex feelings that can occur during dementia diagnosis and journeys.

‘She [Rosa] didn’t actually rest much and I’m not sure that we were very helpful. [...] ‘I sometimes wonder if our negativity […] dampened that strength of hers.’ (Tracey, loved one) (Ethnographic notes 7)

This reflection seemed to aid the processing of thoughts around care transitions for the person with dementia, and the carer experience of that, a process which loved ones valued.

‘It just brought back memories of all the things she wanted to do before she got sick, which was nice.’ (Rupert, loved one)

There were also many positive feelings for the loved ones in sharing memories with staff as they felt a sense of validation and quality interaction.

## Theme 3: Enhanced relationships through self-expression

### Subtheme a: Enhanced relationships between loved ones and members

People with dementia were able to participate on different levels depending on how their dementia presents and how far they have progressed in their dementia journey. StoryTiling provided a medium for meaningful interactions between loved ones and members, and their relationship was enhanced through the meaningful interactions. This took the form of loved ones asking questions, suggesting answers to prompt the member, by physical touch, sharing laughter, or an activity such as walking together. This facilitated a level of closeness or intimacy shared between the loved one and the member regardless of their ability to remember or respond.

Carol begins telling an answer, when she is unsure, she looks to Rupert for assistance and reassurance. When Carol gets a detail incorrect, Rupert steps in and corrects: ‘It’s her daughter actually.’ (Rupert, loved one) ‘Is she?’ (Carol, member) (Ethnographic notes 8.)

The StoryTiling app helps the loved one tell a story that can involve the member. The app provided a structure to the discussion thus propping up the structure of the conversation which appeared valuable to participants.

[Did you have a favourite memory of your dad?] Rosa replies in her own language. Tracey [loved one] translates: ‘He used to travel a lot and then he’d bring back presents.’ (Ethnographic Notes 7)

Simpler levels of participation included mimicking, repeating, nodding along with the reminiscence whilst more active participation where the person with dementia was answering for themselves. The ‘favourite memory’ tile seemed particularly significant in triggering more active participation. The loved ones also placed value on the StoryTiling videos capturing new visualisation of members/guests. The use of StoryTiling to create videos and watch back those videos were impactful on enhancing the relationships between people with dementia and their loved ones.

### Subtheme b: Enhanced relationships between buddies and members

When a buddy was interacting with StoryTiling and a member, they were learning more about the person with dementia. Buddies learn more about member’s life history including their accomplishments, which was then implemented into person-centred care such as targeted activities based on what the member revealed.

‘It helps me to get to know my members as individuals, and to give them the individual support that they need. It gives me ways into creating relationships with people by getting to know fun facts about them, what’s important to them.’ (Scarlett, buddy)

Knowing their history and preferences helped staff creatively respond to the person and try new activities or conversation topics as part of care. One buddy who was a new employee was also able to learn about members/guests by watching the recorded videos on StoryTiling. This reportedly made the buddy feel they got to know the member faster and feel better at their job.

‘If I haven't looked at the video, it might take me quite a while to get to know them. But now I can do straight away. It's like I'm pretty familiar with them easily.’ (Robert, buddy)

Enhanced relationships and deeper understanding between members/guests and buddies occurred through feeling validated by spending time and being listened to.

‘I think that was very kind of you to show interest.’ (Carol, member)

Reminiscence facilitated buddies to gain a deeper understanding of the people they care for. Staff that already knew some things about the members/guests knew them better after a StoryTiling session. This also helped create a full picture of the person, so the buddy can see the individual as unique with lived experience, facilitating respect for the member and their life.

### Subtheme c: Enhanced relationships with other members/guests

The open-plan nature of the environment resulted in other members often walking through the space and sometimes sitting down near the participant. In some cases, this was distracting, yet predominantly, this aided in creating interactions between members that deepened their understanding of each other, showing kindness and compassion. Some members listened to the stories being told and identified with them, thus creating new connections between members.

Another guest/member recognises a name of a place [when listening to another person's story] as well as a football player’s name (Ethnographic notes 6)

“Jim from here, the same thing that killed his mother killed my mother [a Whipple procedure].” (Ethnographic Notes 4).

This demonstrated that storytelling was a shared social engagement, and supported connectedness which was important in the respite and residential settings. This connection between residents was remembered outside the storytelling session on more than one occasion. Theme 4: Person-centred adaptation – the art of the question and trauma informed care.

A key element that arose during the interviews and observations was the method of applying the questions within StoryTiling to the setting for people with dementia. The sessions were predominantly conducted by either a buddy staff member or the insider researcher. These staff were noted to use StoryTiling as a prompt, but then use their knowledge of the person or the loved one to keep the talking going. This active engagement was key, in that the success of the session depended on the facilitator’s effectiveness.

### Subtheme a. The art of choosing questions

There was person-centred adaptation occurring through the process of choosing the question within StoryTiling itself. The person asking questions from the StoryTiling app chose tiles that contained questions they perceived most applicable to the person answering. This was based on their existing knowledge of the person and what they thought the person would be able to answer or how to include both the member and the loved one in a conversation about the member’s life.

Buddy asking questions about working for the Raiders [local football team which they knew was exciting for him to talk about] in relation to Story Tile about career (Ethnographic notes 4.).

These adaptations helped maintain inclusiveness of those contributing to the session, but didn’t stay focussed exclusively on the question; in this manner they honoured the vision to get the conversation going rather than any specific focus to stay ‘on topic’. This respected the level and depth of contribution the person with dementia might make, while including the loved one in the storytelling experience. Similarly in this vein of respect and inclusiveness, questions and tiles were avoided based on perceptions that the answers might be potentially sensitive.

‘[…] but there were some that I thought could upset [Bill], which because I knew him well, I skimmed past’ (Vara, buddy)

When both the loved one and member were participating simultaneously, topic selection was also affected by the person’s desire to create mutual inclusivity. An example of this was when the buddy skipped ‘who was your first love’, being sensitive to the current partner joining the session.

### Subtheme b. The art of knowing the person to prompt the conversation and follow the persons’ lead

It was noted that many of the buddies and loved one used the initial StoryTiling question to open the topic, but then adapted further questions after that. So, the initial question was used as a prompt for the buddy to adapt their own question to suit the needs of the person telling their story.

Many different interviewing techniques were demonstrated throughout the sessions and interviews. Prompting occurred in a person-centred approach and assisted the participant in either primarily accessing the memory or expanding on the story relayed.

‘The questions are helpful for a prompt of what to ask.’ (Isobel, buddy)

This person-centred approach to questioning also helped when guests were thought to be digressing too far from the question asked or seemed stuck on a question.

‘Did you think it was better living here [in Australia after moving from Scotland]?’ (Buddy) ‘I was only very young, but yes I did find friends fairly quickly.’ (Stainless, member)

Interviewers also used the sharing of their own stories as a technique to deepen their connection with the participants. An example occurred when a participant answered a question relating to where he had lived, and the interviewer shared that they had also lived there. They could both reminisce about the town, people and specific places linked to the participants memories, which helped tell more stories.

The buddies used their interpersonal skills to extend the memory or details.

Vara [buddy] telling her own story (Ethnographic notes 3) [when sharing the experience of being raised by their Grandparents].

‘We immigrated from Scotland um, I don’t think I was there. Um. I got out of Scottland when I was about. I think I was… Let me think... um 5 or 6.’ (Stainless, member).’5 or 6? Did you start school in Australia, or…?’ (Sarah, buddy).‘Yes. Primary school.’ (Stainless, member)

In fact, knowing the person helped to maximise reminiscence activity with the aid of the StoryTiling tool. Sometimes buddies continued asking questions that weren’t necessarily relevant to the tile, but this prompted meaningful conversations to flow in an organic way. In many instances, the interviewer wasn’t seeking an exact answer but rather, the questions were used to understand the person and let them tell their stories.

‘I liked that it recorded everything. I liked that it like put them in like a section, even though what came up in the question or what you ended up talking about wasn't actually what the question asked, but like, if you want to go find what you asked you can just go straight in and find it.’ (Sarah, buddy)

This social integration of conversation, supported by the app, was demonstrated in the group setting session, when Michael was telling his story and was stuck on a word in the sentence he was saying, other guests/members suggested words he might mean (Table 4). This aspect illustrated cognitive engagement in the story as well as social engagement in assisting the storyteller in continuing. However, it was noted that when too many people joined, the storyteller appeared reluctant to share as much information. Another limiting aspect was that sometimes the person with dementia would become distressed if they didn’t know the answer, whereas others would respond by talking about a different topic.

‘I think some of the answers went in the wrong sections because he just kept talking. But… that was gonna happen anyway …. You just kind of go off on tangents and that's kind of just what happens.’ (Sarah, buddy)

This lasting impact may have a negative effect as the member checking identified that some people with dementia verbalised their worry with their loved ones after the sessions that they ‘*got some of the questions wrong*’. They were aware of their memory gaps and may have told stories that weren’t how it happened. Similarly, another member asked if he could ‘*go back to that job at the [AIDS] foundation’* that he was talking about in the session; the realisation that the *‘good stuff of the past’* was not the same now was *‘a bit hard’* for the loved one to navigate. The loved ones still highlighted how much they wanted to share the videos and stories with their grandkids, so this negative experience wasn’t the dominant experience however, it is important to note that the StoryTiling sessions have the potential to raise or magnify a sense of loss.

Importantly, the app was seen to be usable and approachable, with an example of a person with dementia seeking to choose which question they want to talk to.

‘Let me find one!’ [question]. The member takes the phone and starts playing with the app themselves, and then starts talking [buddy quickly presses career tile to record him as he starts talking]. (Ethnographic Notes 4)

This positive interaction, and the buddy following the lead of the person with dementia in running the reminiscence session, demonstrated that storytelling and playful use of the questions was more important to them than the recording of those stories.

## Theme 5: Future potential

Other potential uses for StoryTiling were suggested by participants during interviews or observations, and while not as substantial in content were particularly descriptive and potentially valuable.

### Subtheme a. Suggested changes

There were some suggestions to increase the ‘dementia-friendliness’ of the app such as that the questions could be tighter to support shorter answers.

‘I found for people with dementia they're [the questions] a little bit involved. They could have been simpler questions.’ (Isobel, buddy)

Some comments identified that there may need to be some differentiation within the app between dementia care settings and family home settings. Other suggestions related to better video sharing ability, such as having a weblink to share the video outside of the app and sharing the profiles with others not based within the home log in. Potential improvements in functionality of the app were suggested such as being able to more clearly know when recording was occurring and being able to quickly record again after pressing stop.

‘There were a few hiccups like thinking I pressed record, and I hadn’t, and then when Ann started talking about things after the recording had stopped’ (Bob, loved one)

### Subtheme b: Use as a handover tool to support person-centred care

Most responses indicated that the information captured in the StoryTiling app was considered beneficial as a handover tool.

‘[at the hospital] Staff were constantly changing, it was a very difficult time and I thought of the [StoryTiling] app, and thought wouldn’t that have been great for every staff that would come in, to just click on the app.’ (Tracy, loved one).

Although one respondent disagreed with the notion that videos would support nursing care.

‘I think nurses would be too busy to watch a video. I think a quick [section called] about me […], and they could print it and chuck it into their staff file.’ (Vara, buddy)

The value of the handover video was seen if it was concise, but ‘in their own words’ about their preferences and needs, so that staff could get to know the person, and key information to meet their needs. Participants highlighted that there may need to be additional functions like being able to ‘stitch’ videos together with a professional carer to make a '1 minute to learn about me' handover.

‘It would be great if it can be used for the staff as a handover kind of tool as a way to get to know someone. But, it is only useful if the staff actually have protected time to use it, or review the material it captures. Like I have provided a book and a folder, and there are things on her door for staff to get to know her but I can almost guarantee that they don’t have time to read them’ (Bob, loved one). Videos showing the person in their usual state were suggested in being able to contrast with when a person was displaying signs of being unwell.

### Subtheme c: Intergenerational uses

Capturing the life stories was considered particularly useful for future generations by ensuring history was not lost.

‘It'd be good for the family, as in my kids and grandkids to see what was happening in our lives and they may not be aware of all the different trips we've taken or what happened on those trips.’ (Dylan, loved one)

Whilst some loved ones had created life story books for participants, a digital video recording was thought to be more appealing to a younger generation.

‘If somebody fished out a video of grandma sort of giggling about some question, when we're dead and gone they might think oh, that was nice, yes. Now I remember what grandma's like.’ (Rupert, loved one)

Loved ones expressed value in using the stories to keep absent family members updated on their loved ones. However, it was noted that these potential uses were dependent on easy shareability of the videos.

‘We've focused so much on mum's illness and just surviving every day that we haven't really had that contact with them [family]. So they've almost become strangers. […] Contact has been lost. (Tracy, loved one)

### Synthesis of Themes – telling and watching stories support person-centred care

**Figure 3.**
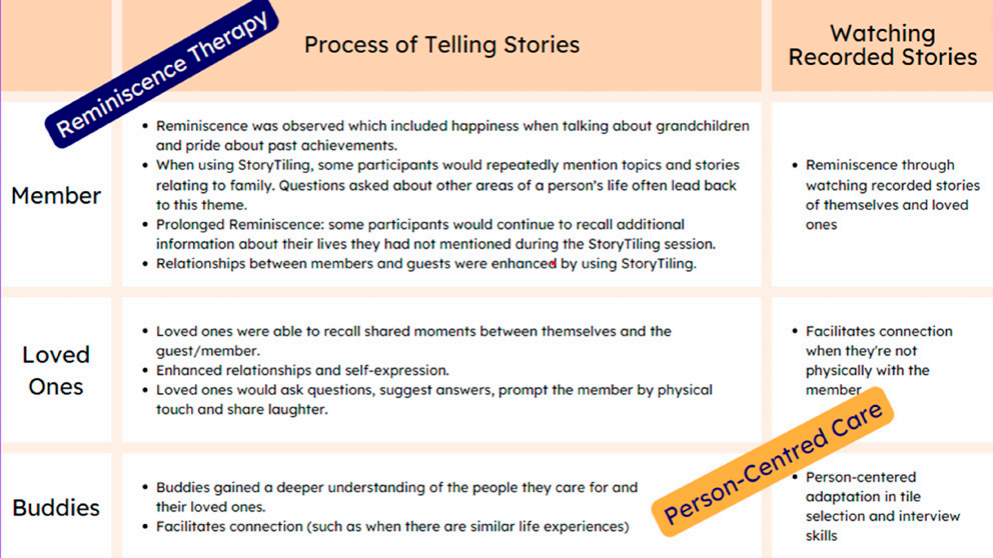
The reinforcing approach of reminiscence therapy and person-centred care using Digital StoryTiling.

The interaction between the themes was noted, whereby the participants could be involved in both the ‘telling’ of stories, and the ‘watching’ of stories. Each kind of participant could have a different interaction with the telling or watching of stories, and these were noted to contribute to function as a reminiscence therapy while also functioning to support person-centred care. The interactive nature and varied use depended on the preferences of the person, family and professional carer, and timing and needs. Figure 3 demonstrates the interaction between telling and watching stories, as reminiscence therapy as well as person-centred care.

## Discussion

This community-based study with 17 participants identified key areas of functionality for StoryTiling in working with people with dementia. Reminiscence was valued, enhanced relationships in multiple ways, was functional in its adaptation to person-centred approaches, and participants identified potential methods for future use.

The act of engaging in reminiscence therapy evoked emotions for guests/members. So-called positive emotions were described or seen, such as enjoyment, happiness, and pride, echoing findings by Ervin et al. (2013) and Karlsson et al. (2014). Other emotions were displayed, such as sadness and anger, and whilst these emotions can be viewed as negative outcomes, access to the range of human emotion is a basic human right (Woods et al., 2018). Dementia can threaten a person’s sense of self (Matthews, 2020; Platel et al., 2021) but is impacted by feedback from others based on the person’s perceived value and purpose. StoryTiling provided a mechanism to reinforce the person’s sense of self through interactions and memory telling with other members/guests, loved ones and buddies which lasted beyond the interview episode. The cyclical nature of the storytelling, which enabled ongoing revisiting of the topic and incorporation into the buddy’s knowledge of that person, emphasised the personality and core elements of that person’s identity and character. Sharing stories makes a person’s life more known and visible (Witham & Haigh, 2018), enhancing their sense of self and wellbeing.

Prolonged and repeated reminiscence can enhance both the quality of memories, and the ability to recall them (Platel et al., 2021) and likely increases the gain of global cognitive function achieved through reminiscence (Kirk et al., 2019). Participants in this study continued to reminisce after the research intervention. They recalled details they had previously forgotten, suggesting it became a prolonged and repeated activity leading to potential cognitive improvement and sense of identity.

Using the StoryTiling app has potential benefits for loved ones, as reminiscence therapy for older people has been shown to enhance self-esteem and promote happiness and feelings of wellbeing, significantly reducing symptoms of depression (Tam et al., 2021). Further research on digital story telling could investigate the impact on self-care, social and leisure activities and health outcomes for carers (Engel et al., 2022; Mohanty & Niyonsenga, 2019).

Remembering the person before dementia was facilitated by the reminiscence activity. Ryan et al. (2020) described this joint reminiscence as *“enabling carers to see the person within the dementia rather than the dementia within the person”*. Whilst this reminiscence is evocative of a range of emotions, StoryTiling as a reminiscence tool prompted memories of shared moments, helped loved ones learn stories they had not known previously and facilitated celebration of life. This is consistent with Ryan et al. (2020) and often appeared to elicit feelings of fondness between the loved one and the member. This included the varied ability of the person with dementia to remember or respond. Ryan et al. (2020) suggested that reminiscence was necessary for establishing and or improving connections by redefining them as relationship-focussed. A person’s sense of self persists whilst experiencing dementia (Platel et al., 2021) and self-confidence is gained through relationships with others founded on respect and understanding, and feedback on the persons value and purpose (Matthews, 2020). Being known by others, in this instance by sharing stories with other members, facilitates retention of sense of self and identity.

The digital reminiscence tool enhances person-centred care (Ryan et al., 2020) as it enabled buddies to gain a deeper understanding of the people they care for. Outcomes of person-centred care are well documented in literature and include reductions in behavioural symptoms of dementia (Isaac et al., 2021), higher quality of care (Chenoweth et al., 2022; Tsai et al., 2015), and decreased mortality (Meterko et al., 2010). Collaborating with and involving families brings expert knowledge about the person’s life history and their current preferences and helps build meaningful, respectful relationships, resulting in enhanced capacity to deliver person-centred care (Haunch et al., 2021).

The practice of StoryTiling reminiscence was found to reinforce this previous research with a clear and implementable method. StoryTiling increased the staff’s knowledge of a person’s life stories, which participants advocated as enhancing person-centredness. This is in keeping with previous research where staff identified technology enhanced reminiscence therapy allowed them to engage with the person, gain a deeper understanding of them as a person and drive personalised care (Goodall et al., 2021). There was also promise for the app to support continuity of care, which focusses on staff getting to know the person more deeply and maintaining contact over time (Lei et al., 2021), rather than continually changing staff members, which can be common in aged care settings with high casualised workforce (Gibson, 2022). Having a digital tool where staff and families are supported to engage in focussed activities of reminiscence supports rapid development of deeper relational knowing of a person living with dementia, which can also support satisfaction with quality-of-care delivery for staff, a known component of burnout (Westerman et al., 2014).

Consideration of potential trauma is essential in dementia care, as age related deterioration occurs earlier in those who have previously experienced trauma (Craftman et al., 2020). Similarly, cognition is affected by post-traumatic stress disorder resulting in increased risks for developing dementia (Burri et al., 2013), and re-traumatisation can exacerbate the symptoms of dementia (Sweeney et al., 2018). All staff/buddies at the participating organisation receive training in trauma-informed practice, and adapted questions based on their knowledge of the person and potential trauma triggers. Various communication techniques were used throughout the sessions, including active listening, prompting, redirecting, rephrasing and communication of shared experiences. While StoryTiling provides the starting point through questions, and knowing the person enhances the reminiscence activity, interviewing techniques and skills are needed for active engagement and to elicit or enhance stories. More often than not, participants were redirected back to the conversation by the person with dementia or their loved one, indicating that awareness but not complete avoidance of trauma triggers was practised in this case, and following the lead of the person with dementia (Craftman et al., 2020; Han et al., 2021; Sweeney et al., 2018). Recognising the person’s agency and choice in reminiscence was demonstrated by sensitively following the conversational lead of the person with dementia. Deeper, more enhanced stories result in the ability to understand the person in a cultural context (Witham & Haigh, 2018), contributing to an improved capacity to deliver person centred care.

The person-centred, sensitive, and trauma-informed approach by staff to implementing StoryTiling may have been particularly related to the workplace culture. Buddies are employed with guests/members helping choose potential workers through interviews and engagement (D’Cunha et al., 2023). The emphasis of the organisational philosophy is on individualised care and a ‘never say no’ approach to supporting people living with dementia’s requests for activities. Environmental settings play a role in implementing technology and reminiscence (Barbosa et al., 2015; Travers et al., 2016; Yevcheck et al., 2017) and would be an important consideration for future uptake of the digital reminiscence tool.

Many digital formats need to be accessible for staff who are time-poor. This study highlights that using reminiscence as an activity may provide a ‘two-for-one’ process of staff learning for person-centred care and therapeutic interaction for the person with dementia. Using the information in a handover could be valuable, and the video format could potentially minimise the impact of cultural and language barriers present in aged care staff (Al Shamsi et al., 2020; Amoah et al., 2019; Bello, 2017). Up to 91% of direct support workers are from culturally or linguistically diverse backgrounds (AIHW, 2021), so easily accessible person-centred approaches are needed. There may also be some aspects of these findings which can be easily translated into practice for staff working with the dementia population: for example, the ‘favourite memory tile’ and ‘how do other people describe you/your loved one’ seemed to feature favourably with participants, and can be used as conversation starters without the app. Future research could examine whether a free app supports this, or whether these kinds of conversation starters could be more easily embedded into aged care practice in other ways. It may be that an app offers a form of modelling to enable care workers to engage in this style of engagement, but more work is needed to understand the socialisation and potential.

## Strengths and limitations

Strengths of this study are the dual qualitative approaches of ethnographic and interview data collection and analysis, allowing for in in-depth understanding of complex phenomena. Limitations are the tensions between the ‘insider researcher’ approach, outlined in the method section. As qualitative research, this study is not seeking generalisability. It is worth noting that the sites hosting the intervention were already very person-focussed; trials in larger and institutional sites would be beneficial to understand transferability to settings that may be more institutional in their timetable and activity design.

## Future research

Ethnographic observations became difficult if the researcher was also the person operating the app, asking the questions and recording the videos therefore, it is recommended that the researcher observe another person using the StoryTiling app. . Incorporating training in interviewing technique could be tested as providing potential benefit for engaging participants and eliciting their stories. Recent research reported that prompting memories using words was not effective (Kirk et al., 2019)**,** whereas objects, photographs (Cuevas et al., 2020), and music (Platel et al., 2021) were effective. Further research could include these as adjuncts whilst using the StoryTiling app. Further developing the app’s capacity to create a short video/resource to support person- centredness in handovers could be investigated; as would implementation into a larger residential aged care setting with greater complexity of staffing. These investigations would further advance the applicability of the free digital reminiscence therapy as an adjunct to person-centred care.

## Conclusion

StoryTiling, as a digital reminiscence tool, was effective in aiding person-centred dementia care. A digital tool enabling reminiscence supports rapid development of deeper relational knowing of a person living with dementia and can respond to the complex health needs of older Australians and healthcare workforce to deliver timely person-centred care. The process was dependent on the ‘art of the question’ and the ‘art of the interview’, particularly by people who know the person with dementia and are trauma-informed in order to progress interviews and utilise them within the care environment effectively.
